# Fatigue in adults with Osteogenesis Imperfecta

**DOI:** 10.1186/s12891-019-3000-7

**Published:** 2020-01-03

**Authors:** Arjan G. J. Harsevoort, Koert Gooijer, Fleur S. van Dijk, Daniëlle A. F. M. van der Grijn, Anton A. M. Franken, Anne Marieke V. Dommisse, Guus J. M. Janus

**Affiliations:** 1 Expert Center for adults with Osteogenesis Imperfecta, Isala Hospital, Zwolle, The Netherlands; 2 North West Thames Regional Genetics Service, Ehlers-Danlos Syndrome National Diagnostic Service London, North West Health Care NHS Trust, Harrow, Middlesex, UK

**Keywords:** Osteogenesis Imperfecta, Fatigue, Fatigue severity scale

## Abstract

**Background:**

Osteogenesis Imperfecta (OI) is characterized by bone fragility, and features such as blue sclerae, dentinogenesis imperfecta, hearing loss, ligamentous laxity and short stature can be present. It has long been assumed that the functional ability and quality of life of patients with OI depends primarily on the severity of skeletal deformities. However, fatigue is often mentioned in clinic by patients with all types of OI as an important modifier of their quality of life and does not always seem to be related to their functional ability. The aim of this study is to investigate whether adults with Osteogenesis Imperfecta are significantly more fatigued than the normal population.

**Methods:**

The Fatigue Severity Scale (FSS) was distributed by mobile phone application among 151 adult patients with different OI types. Results of the FSS in the OI group were compared with two control populations from America (*n* = 20) and the Netherlands (*n* = 113).

**Results:**

Ninety-nine patients (OI type 1 (*n* = 72), OI type 3 (*n* = 13), OI type 4 (*n* = 14) completed the FSS questionnaire. The mean FSS score of this cohort was 4.4 and significantly higher than the control populations (2.3/2.9). 65% of our cohort reported at least moderate fatigue compared with 2 control populations from America and the Netherlands.

**Conclusion:**

Fatigue in patients with OI is a frequently encountered problem in our expert clinic but research into this topic is sparse. This pilot study is the largest study to date investigating fatigue in patients with OI and results have been compared with two control groups. The mean FSS score of 4.4 in the OI group indicates that people with OI are generally significantly more fatigued than the control population. Further evaluation of fatigue and its influencers in a larger group of OI patients is important for future management.

## Background

Osteogenesis imperfecta (OI) is a rare hereditary disorder with a prevalence of 6–7:100,000 [1]. OI is primarily characterized by bone fragility. Additional features of OI include blue sclerae, dentinogenesis imperfecta, hearing loss, ligamentous laxity and short stature [2–6]. OI is known to be a clinically variable disorder with severity ranging from perinatal lethality to slightly increased fracture frequency with normal life expectancy [3]. As such, the clinical classification of OI consists of 5 different types (1–5) [6] In approximately 90% of patients with OI, dominant mutations in the genes *COL1A1* and *COL1A2* encoding respectively the alpha1 and alpha2 chains of the protein collagen type I, are identified [6]. The functional ability of patients with OI, especially ambulation, have been historically attributed to the severity of the skeletal deformities [3, 6] and this has long been the focus of physicians involved in the care of patients with OI. However, many patients visiting our expert center for adults with OI complained about fatigue, which limits their quality of life, and asked whether this could be related to their diagnosis of OI. Previous studies indicate that the quality of life (QoL) of individuals with OI is negatively influenced by reduced function due to fatigue indicating that fatigue is an important factor when considering quality of life in OI patients [7–10]. As such, we approached a subgroup of our total group of OI patients to investigate the impact of fatigue on daily functioning compared to control populations.

## Methods

### Study design and population

A cross-sectional cohort study was undertaken in the national expert center for adult patients with Osteogenesis Imperfecta, Isala Hospital, Zwolle, The Netherlands. All patients who visited the expert center from December 2007 until December 2015 were selected to participate. The main exclusion criteria were unreturned questionnaires. Informed consent was obtained from each participant. The study was registered in the Isala research registry (Nr.190106) and the local Medical Ethical Committee approved the study protocol and granted an exemption because participants are not subject to procedures and are not required to follow rules of behavior.

### Data collection

Many definitions of fatigue exist [11] as well as scales to measure the nature, severity and impact of fatigue in a range of clinical populations [12]. To investigate fatigue in patients with OI the Fatigue Severity Scale (FSS) was distributed among all adult patients. The FSS questionnaire is widely used and has been found valid and reliable in different patient groups [13] It is developed to measure the impact of fatigue on daily functioning [14] and consists of the following nine statements: 1. My motivation is lower when I am fatigued. 2. Exercise brings on my fatigue. 3. I am easily fatigued. 4. Fatigue interferes with my physical functioning. 5. Fatigue causes frequent problems for me. 6. My fatigue prevents sustained physical functioning. 7. Fatigue interferes with carrying out certain duties and responsibilities. 8. Fatigue is among my three most disabling symptoms. 9. Fatigue interferes with my work, family, or social life. The higher the score (on a scale of 1–7), the higher the impact on fatigue in daily living (1 completely disagree, to 7 completely agree.)

The questionnaire was sent to the patients in the form of an email containing a link to download a mobile application. If participants were unable to download the application, the questionnaire was sent by email or regular post. To assess how fatigue influences daily living in OI patients we analyzed the distribution of scores for the 9 separate statements. The severity of fatigue was calculated as a mean FSS score of all nine items per patient ranging from 1.0 (no fatigue) to 7.0 (maximum fatigue).

Medical records were analyzed from patients who completed the FSS to determine gender, age and the type of OI according to the updated Sillence criteria [3]. Means and standard deviation (SD) were given for normally distributed continuous variables. Differences in means comparing OI patients and separate FSS questions were tested using independent t-tests and the mean differences were presented as the mean with 95% confidence intervals (95%CI). A two-sided *p*-value of 0.05 was considered significant. All data were analyzed with SPSS (statistics 24.0.)

### Control populations

To evaluate the impact of fatigue on daily living in OI versus controls, we compared the FSS scores from our cohort with two previous studies that used the FSS. The first study by Krupp et al. 1989 [14] investigated fatigue in individuals with MS (multiple sclerosis) and SLE (systemic lupus erythematosus) and in a control group consisting of 20 healthy American individuals selected from volunteers unfamiliar with the study with a mean age of 39.7 years SD 9. The American control group scored a mean of 2.3 SD 0,7. The researchers determined a cut off score > 4 for severe fatigue, influencing daily living [14]. The second study concerned the study of Merkies et al. 1999 [15] which investigated fatigue in immune-mediated polyneuropathies and recruited a Dutch control group (*n* = 113) from hospital personnel, companions (relatives, friends) of patients visiting their outpatient clinic, and volunteers unfamiliar with their study. These patients declared themselves to be healthy, free from any chronic medical condition, and were not taking medication that could contribute to fatigue. This control group consisted of 54 men and 59 women with a mean age of 54.2 (range 18–83) being an average cohort out of the Dutch population and comparable to our OI cohort regarding age and gender distribution. The Dutch control group had a mean and median FSS of 2.9, SD 1.1. Severe fatigue was defined as FSS score > 5.1 (mean + 2SD) and fatigue was defined as FSS score > 4 (mean + 1SD, *n* = 113, 15].

## Results

### Clinical characteristics

We approached 221 OI patients who had visited the expert center to participate in this study and to fill in the questionnaire. The age range of this cohort was 18–80 years. Permission and signed informed consent were received from 151 patients. A group of 52 patients did not complete the questionnaire and was therefore excluded. Therefore, 99 patients (65.1% response rate) were available for analysis. It concerned individuals with type 1 (*n* = 72), type 3 (*n* = 13) and type 4 (*n* = 14). Sixty-one women and 38 men were included. The mean age was 45 (age range 19–80 years). These distributions are comparable to our total OI population [16].

### Fatigue severity score

#### Participant basic characteristics and total scores

The mean and median FSS score of the individuals with OI in our cohort were respectively 4.4 and 4.8, SD 1.4 (95% CI 4.16–4.70). According to the Kolmogorov-Smirnov test, the distribution of the FSS mean score was normal (*P* = 0.105). 42% (*n* = 42) of the respondents had a mean FSS score of ≥5 whilst 23.1% (*n* = 23) had a mean FSS score between 4 and 5. The man/woman distribution in the cohort was 40.5% (*n* = 17)/ 59.5% (*n* = 25).

A single sample t-test and the Mann-Whitney U test were conducted to determine if the differences between the FSS score in the OI group versus the American and Dutch controls were statistically significant, concluding that individuals with OI in this cohort have statistically higher fatigue scores than the American control group, t (98) = [15.46], *p* = [0.000], and the Dutch control group, t (98) = [11.10], *p* = [0.000].

Statements 3 and 4 of the FSS had both higher median scores with a smaller 95% confidence interval of the mean (4.63 CI 4.27–4.99 and 4.66 CI 4.32–4.99) (significance 0.099, 0.067) compared to the other questions. Statements 6 and 8 had also a high median score (4.23, 4.67), but overall more diffuse results as can be seen in the 95% confidence interval (3.86–4.7; 4.22–5.12) (Table 1).

**Table 1:** Mean score per FSS statement for the whole OI group and according to gender

FSS Statements	Mean score	95% Confidence interval	Mean men	Mean women	Difference gender signific. (independent t-test)
1	5.43	5,14-5,59	5.47 ± 1.67	5.41 ± 1.38	0.837
2	4.16	3,82-4,5	4.03 ± 1.76	4.25 ± 1.69	0.538
3	4.63	4,27-4,99	4.21 ± 1.99	4.89 ± 1.63	0.069
4	4.66	4,32-4,99	4.58 ± 1.87	4.70 ± 1.56	0.719
5	3.71	3,36-4,06	3.66 ± 2	3.74 ± 1.6	0.836
6	4.23	3,86-4,7	4.16 ± 2.4	4.36 ± 1.92	0.661
7	4.14	3,75-4,53	3.89 ± 2.12	4.3 ± 1.82	0.320
8	4.67	4,22-5,12	4.08 ± 2.42	5.03 ± 2.08	0.048
9	4.19	3,77-4,62	3.87 ± 2.26	4.39 ± 2.04	0.338
Total	4.43	4.16–4.7	4.22 ± 1.57	4.56 ± 1.22	0.234

#### Gender differences

Table 1 shows that there were no significant differences per gender with regard to the total FSS score. Women scored higher (4.56 ± 1.22) than men (4.22 ± 1.57) on the total FSS score and also in all separate statements except statement 1. On statement 8 this difference was significant. (w:5.03 ± 2.08, m:4.08 ± 2.42 (*p* = 0.048).

#### Age group distribution

**Fig. 1:**
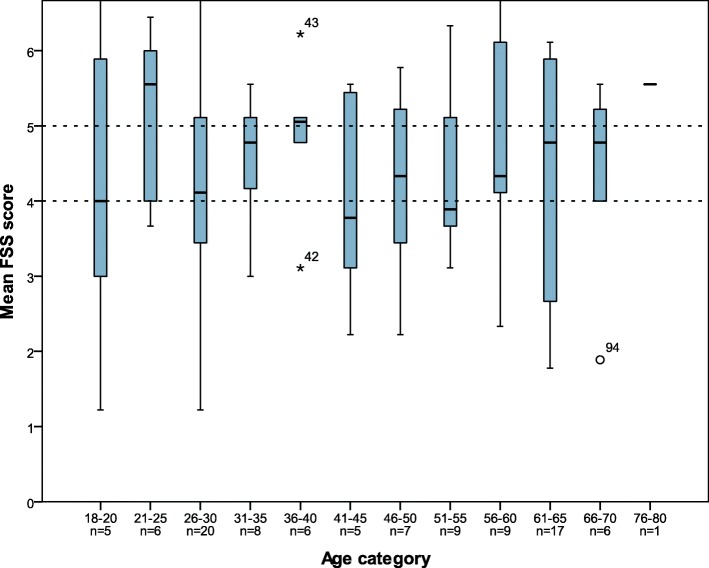
FSS score per age with marking of moderate and high fatigue scores according to Krupp et al

A visual comparison of the separate FSS scores between the different age categories is shown in Fig. 1. The FSS score for question 1 in age category 41–45 is significantly lower (2.4) than the remainder age categories in our study cohort (5.8). (independent T-test *p* = 0,000). All other comparison did not reveal significantly different values.

#### Differences between types of OI

There were no significant differences per OI type for the mean FSS score. The FSS mean scores were in all OI types ≥4 (Table 2).

**Table 2:** Mean total FSS score per OI type

	Mean FSS score	Standard Deviation
OI type 1 *N* = 72	4.38	1.36
OI type 3 *N* = 13	4.33	1.69
OI type 4 *N* = 14	4.75	1.14

There were no significant differences per OI type for the separate FSS statements (data not shown). In the group with a mean FSS ≥5, the distributions regarding OI type were: OI type 1: 64.3% (*n* = 27), OI type 3: 13.4% (*n* = 6), OI type 4: 21.4% (*n* = 9). People with OI type 4 scored higher than people with OI type 3 and OI type 1 on question 3, 6 and 8.

## Discussion

Fatigue is often mentioned by individuals with OI during the clinical appointment. As the prevalence and experience of fatigue in patients with OI is largely unknown, we set out to perform a pilot study regarding occurrence and severity of fatigue in people with OI to determine whether this needs to be explored further. 99/151 patients filled in the FSS. We assessed the medical records for age, gender and type of OI. We did not analyze for any medical confounders such as recent fracture(s), cardiac or lung complications, initiated therapy, physical exertion, mobility and work. The mean and median FSS score of the individuals with OI was respectively 4.4 and 4.8.

### FSS results compared to results in two control groups

The fatigue scores in our study cohort are significantly higher compared to the Dutch national control group(*n* = 113) [15] and the American control group (*n* = 20) [14]. Merkies et al. [11] define a mean FSS score ≥ 5.1 as severe fatigue, and a score > 4 and < 5 equates “borderline fatique “[15]. When analysing the FSS results of the OI cohort according to the definitions of Merkies et al. the OI cohort experiences borderline fatigue, influencing daily living, with regard to the mean FSS score.

Krupp et al. [14] defined a FSS score of > 4 as moderate to high fatigue level, influencing daily living. When analyzing the FSS results according the definition of Krupp et al. [14] it appears that 42.4% of the respondents (*n* = 42) had a mean FSS score of five or higher indicating severe fatigue. 23.1% (*n* = 23) had a score between four and five indicating borderline fatigue. When analysing the results with the definition of Merkies et al., it appears that 38.4% of the respondents (*n* = 38) had a mean FSS score of five or higher indicating severe fatigue. 27.3% (*n* = 27) had a score between 4 and 5.1, indicating borderline fatigue. These mean FSS scores are very high compared to the general population, with only 5% of the general population being severely fatigued [15]. The presence and severity of fatigue is almost equal across all OI types, which could indicate that OI type and severity of OI is not influencing fatigue. This may demonstrate that although most people with OI type 1 will have reached a higher level of daily functioning than patients with OI type 3 and 4, they still experience comparable impact of fatigue on their daily functioning. The FSS scores in the OI cohort also exceed minimal clinically important difference (MCID) values determined for other patient groups, which are for example 0.4 for SLE and 0.7 for RA (rheumatoid arthritis) [17, 18]. Given the above, there appears to be sufficient evidence for the presence of increased occurrence and severity of fatigue in OI patients in the investigated cohort.

### FSS results compared to one similar study involving OI patients

A comparable study was recently performed in Norway by Arponen et al. [9]. It concerned a cross-sectional study of responses of OI patients matched with healthy controls from Norway to a questionnaire, designed to evaluate levels of experienced fatigue and body pain as well as presence or absence of symptoms related to sleep disturbance or sleep apnoea. Fatigue was evaluated with, among others, the FSS questionnaire which demonstrated a FSS mean score of 5 in patients with OI(*n* = 56). Interestingly, the Norwegian control group scored a mean FSS score of 4 (*n* = 56). Arponen et al. concluded that in comparison with age and gender matched controls, adults with OI do not differ in experienced fatigue [9].

The Dutch control group [15], has a lower mean FSS score (2.9, *n* = 113) than the control group in the Norwegian study of Arponen et al.(4.0, *n* = 56, 9]. Compared to the American original validation [14] who report a mean FSS of 2.3 ± 0.7 (*n* = 20) again the mean FSS score in the Norwegian control group is high.

However, there may be an explanation for the high score in the control group as a Norwegian national study investigating fatigue in the general population, [19] concluded that the high FSS scores in the general population of Norway can be due to difficulties in translation of the US-English version of the FSS into Norwegian because of lack of the concept of fatigue in Norwegian language [19]. A valid comparison between Norway, the Netherlands and the US regarding the FSS may therefore not be possible. A validation of the FSS in a Swiss control group is comparable to the Dutch and American results with a mean FSS score of 3.00 ± 1.08, (*n* = 454) [20]. As such, we can conclude that the mean FSS score of our Dutch control group is comparable with the American and Swiss control groups and that our earlier conclusion that the severity of fatigue is increased in the Dutch OI cohort still holds true.

### Limitations of this study and further directions for research

There is a low response rate (151/221 gave consent and 99/151 filled in the FSS) when looked at the initially approached patients. It is difficult to speculate why this could be the case but an important factor may be that with regard to consent as well as with regard to filling in the FSS, patients were only approached once and were not sent reminder(s). Biases are difficult to avoid as it may be that the people who felt that fatigue was influencing there life significantly, were more inclined to participate but it is also possible that these patients were limited by fatigue to participate in the study. As mentioned before, there are many scales to measure the nature, severity and impact of fatigue in a range of clinical populations and a limitation of the FSS is that it is a general questionnaire, and as such not specially developed for OI. The FSS however explores the severity of fatigue and is therefore suitable for initial screening in different clinical populations and can be used for longitudinal measurements which is important in assessing whether fatigue can increase or decrease over time and exploring possible modifiers of fatigue. Another limitation of our study lies with the control populations as both the Dutch control group and the US control group date from respectively 1999 and 1989 and trends in fatigue may change in the population over time.

Lastly, we did not investigate any factors that influence fatigue in OI patients in our study, but this is an important direction for further research into fatigue in patients with OI as fatigue may influence QOL. Other factors have been reported as well [21]. It is already known that the presence of pain, but also educational level and employment status influence the severity of fatigue. Bathmen et al. published on fatigue in Marfan syndrome, another hereditary connective tissue disorder. The authors concluded that occurrence of chronic pain and employment status influenced the severity of fatigue [22]. Interestingly, a study in children with OI reported a decrease of the level of fatigue after a 12-week individual and supervised physical training program, and increase of the level of fatigue after the program had stopped [10, 23]. Studies in other patient groups, including people with Marfan syndrome reported good effects of physical activity on fatigue [24–26]. This is important knowledge since some OI patients or parents of OI patients tend to limit their physical activity when they become aware of the inherited bone fragility [23]. Some age categories may benefit from an individual and supervised training program.

## Conclusion

In this study the influence of fatigue on daily functioning was investigated in the largest cohort of OI patients to date and compared with control groups in particular a national control group. Although, there were several limitations of our study, based on the current data, there is sufficient evidence for increased severity of fatigue in our cohort of OI patients. An important direction for future research is performing longitudinal measurements using the FSS and exploring determinants of fatigue as this may be of importance for the quality of life in OI patients.

## Data Availability

The datasets used and analysed during the current study are available from the corresponding author on reasonable request.

## Abbreviations

95%CI: 95% confidence interval
FSS: Fatigue severity scale
MCID: Minimal clinically important difference values
MFS: Marfan syndrome
MS: Multiple sclerosis
OI: Osteogenesis Imperfecta
QOL: Quality of life
RA: Rheumatoid arthritis
SD: Standard deviation
SLE: Systemic lupus erythematosus
